# Prevalence of exposure to someone else’s firearm violence, threats, and risky behavior among a national sample of young people in the United States

**DOI:** 10.3389/fpubh.2025.1451268

**Published:** 2025-04-03

**Authors:** Kimberly J. Mitchell, Victoria Banyard, Bruce G. Taylor, Elizabeth A. Mumford, Weiwei Liu, Heather A. Turner

**Affiliations:** ^1^Crimes Against Children Research Center, University of New Hampshire, Durham, NH, United States; ^2^Department of Social Work, Rutgers University, New Brunswick, NJ, United States; ^3^NORC at the University of Chicago, Chicago, IL, United States

**Keywords:** firearm violence, bystanders, mental health, intervention, prevention, gun violence, suicide

## Abstract

This article provides prevalence rates for a wide range of types of exposure to someone else’s firearm violence, threats, and risky behavior among youth and young adults across several different demographic and structural characteristics. Data are from the *Growing Up with Guns* study of 5,311 participants recruited through the AmeriSpeak Panel. Data were collected from September 2023 to January 2024. Eligibility included U.S. residents who were ages 10–34 years old and proficient in either English or Spanish. A majority of participants (69.5%) reported exposure to someone else’s firearm violence, threats, or risky firearm behavior—either interpersonal or self-directed—in their lifetime. Rates of exposure were high across age categories, ranging from 48.0% of 10–17 year olds to 80.3% of 25–34 year olds. Odds of exposure also varied by race, sexual minority identity as well as deficits in social determinants of health (SDOH). Such wide-spread exposure introduces opportunities to prevent shootings before they occur and can inform the development of bystander interventions targeting those who are witnesses or otherwise know about another person’s firearm violence, threats or risky behaviors.

## 1 Introduction

Dozens of leading professional organizations, including the American Bar Association and the American Medical Association, have endorsed a public health approach to gun violence prevention (1). Central to a public health approach is the early identification of situations that may lead to gun violence and understanding how and when people hear about it. Bystanders may play an important role—they are third parties who witness violence, who may know about students carrying firearms to school, or who may know about someone’s plan to use a firearm for violence as expressed on social media. Violence prevention initiatives increasingly involve these third parties in prevention training, seeing them as potentially important gatekeepers or facilitators in prevention efforts. There is already evidence that bystander action can reduce intimate partner violence, sexual assault, stalking, and bullying in young adults and adolescents (2–11). And, most recently, there is increasing attention to bystander action as a possible youth violence prevention strategy (12).

We know very little about this approach as a possible prevention tool for situations involving risk for gun violence. However, given the success of bystander-focused prevention approaches for reducing other risk behaviors and changing social norms toward anti-violence (13), a logical next step is to explore the roles that bystanders might play in relation to reducing risky firearm use (14). To do that, we first need to understand the context and frequency of youth and young adults’ exposure to someone else’s gun violence, threats and risky behaviors using general population samples that are national in scope. We also need to better understand how social locations, including aspects of social identity and features of community spaces where young people live, relate to their likelihood of gun violence exposure, as that may help determine how relevant they may perceive such prevention efforts.

### 1.1 People can be exposed to firearm violence, threats, and risky behaviors in a variety of ways

Researchers note that the field of firearm violence research has most often focused on direct victims and perpetrators of firearm violence and outcomes including fatalities and injuries (15). These are critical public health questions. However, a growing body of work has begun to examine the negative collateral effects of exposure to firearm violence beyond direct victims and perpetrators (15). This has at times involved limited definitions of exposure, such as studies using census tract rates of firearm fatalities (16). Indeed, recent studies are moving beyond defining exposure as seeing, hearing or knowing someone who has been shot to understanding exposure to risky firearm use and exposure to threats of firearm use as well (17). Bancalari’s review describes a broader variety of ways exposure has been operationalized. Specifically, seeing gunfire is one main way exposure is assessed with self-reports, with some studies also adding hearing gunfire. A few studies also include knowing someone who was the victim of firearm violence, knowing someone who carries a gun, or general levels of awareness of gun violence in one’s community. Bancalari and colleagues summarized three important categories of exposure: bystanders (who hear or witness shots or witness someone being threatened with a gun), vicarious exposure (knowing someone who was shot or knowing someone exhibiting risky gun carrying), and community (awareness of gun violence in one’s neighborhood). Lennon and colleagues separated indirect exposure (hearing gunshots or knowing someone who was a victim) and direct (witnessing a shooting, being threatened with a gun, and being a victim directly by being shot or injured). While this measure included threats, it also confounded victimization of oneself along with witnessing a shooting as part of the same category. Beseler and colleagues (18) also included exposure to someone using guns for self-directed violence. Quimby and colleagues (19) expanded this topic to focus on exposure to guns (rather than violence) to include a variety of dimensions of gun access as a risk factor. Researchers call for the continued development of broader measures of exposure to firearm violence (15), including understudied dimensions like exposure to guns and someone else’s self-directed gun violence, and exposure to threats of gun violence. The current study sought to examine a broader set of firearm exposure items in a national general population sample.

### 1.2 The role of social determinants of health in understanding exposure to firearm violence

Firearm exposures are also unequally distributed across locations, with communities bearing the adversity burden of greater deficits in social determinants of health (SDOH) also experiencing greater firearm violence exposure (16). Studies of firearm violence broadly, and exposure specifically, are also turning to community models of risk and resilience (20). These models note constructs beyond the individual that may influence exposure risk and suggest innovative prevention and intervention strategies. This is consistent with calls to study gun violence exposure within models of systematic inequality (15). Measuring SDOH is one strategy for enhancing models of risk and protective factors. SDOH are conditions in the places where people live, learn, work, and play that affect a wide range of health and quality-of life-risks and outcomes, including mental health (21). There is a growing awareness of the importance of including assessments that will capture and integrate such conditions into public health research. Such measurement permits analysis of the causes and conditions of differing rates of public health problems and helps overcome the limitations of assessing only demographic markers (22). Specifically, SDOHs help identify modifiable factors that can be the object of policy and program interventions to prevent gun violence. There is also a growing knowledge base about the importance of SDOHs across the social ecology in relation to mental health that shows risk for distress related to low income, unemployment, neighborhood problems, social identity group membership (23, 24).

### 1.3 Current study

The current study provides prevalence rates for a wide range of different situations involving exposure to firearm violence, threats of firearm violence, and risky firearm behavior (e.g., inappropriate carrying or possession) among youth and young adults across several different demographics and SDOH characteristics. The current study builds on previous work by analyzing sub-types of exposure—firearm violence, threats, and risky behaviors—reflecting contextual SDOH measures using a nationally representative general population sample covering a wide developmental age range.

## 2 Materials and methods

### 2.1 Participants

Data were collected from 5,311 youth and young adults who were part of the AmeriSpeak panel for the nationally representative *Growing up With Guns* Study, an often-used panel for health research (25–28). The survey was administered from September 2023 to January 2024. From the panel households, individual residents who were eligible to participate in the study were youth or young adults, ages 10–34 years old, who can speak or read either English or Spanish. Demographic details of the weighted sample are in Table 1.

**Table 1:** Sample demographic characteristics by report of any exposure to firearm violence, threats or risky behavior.

Characteristic	All participants n (%)	No firearm violence exposure n (%)	Any firearm violence exposure n (%)	*p* value
All participants	N = 5,311	1,410 (30.5)	3,901 (69.5)	–
Demographics				
Age				
10–17	1,189 (30.2)	551 (52.0)	638 (48.0)	<0.001
18–24	853 (28.2)	168 (19.7)	685 (80.3)	
25–34	3,269 (41.6)	691 (22.1)	2,578 (77.9)	
Mean age				
Sex assigned at birth^a^				
Female	3,282 (49.6)	791 (28.1)	2,491 (71.9)	<0.001
Male	1908 (47.9)	559 (31.9)	1,349 (68.1)	
Intersex	17 (0.3)	3 (12.5)	14 (87.5)	
Prefer not to answer	104 (2.3)	57 (55.1)	47 (44.9)	
Gender identity^a^				
Cisgender female	3,174 (47.4)	784 (29.3)	2,390 (70.7)	<0.001
Cisgender male	1870 (47.1)	559 (32.5)	1,311 (67.5)	
Gender minority	166 (3.6)	29 (14.9)	137 (85.1)	
Missing	101 (1.9)	38 (38.3)	63 (61.7)	
Sexual identity				
Heterosexual	4,264 (81.7)	1,253 (33.4)	3,011 (66.6)	<0.001
Sexual minority	1,047 (18.3)	157 (17.3)	890 (82.7)	
Race^a^				
White	2,803 (62.9)	812 (33.0)	1991 (67.0)	<0.001
Black or African American	946 (14.2)	176 (21.2)	770 (78.8)	
Asian	535 (7.1)	178 (34.5)	357 (65.5)	
American Indian or Alaska Native	63 (0.8)	9 (15.3)	54 (84.7)	
Native Hawaiian	20 (0.4)	7 (34.0)	13 (66.0)	
Other race	281 (4.1)	66 (25.7)	215 (74.3)	
Two or more races	523 (7.5)	105 (23.4)	418 (76.6)	
Prefer not to answer	140 (3.0)	57 (39.2)	83 (60.8)	
Ethnicity				
Not Hispanic or Latino	4,149 (77.6)	1,077 (29.6)	3,072 (70.4)	0.16
Hispanic of Latino	1,104 (22.4)	305 (32.7)	799 (67.3)	
Structural and Social				
Annual household income^a^				
Less than $30,000	1,273 (22.8)	305 (28.8)	968 (71.2)	<0.001
$30,000 to under $60,000	1,373 (24.2)	331 (25.5)	1,042 (74.5)	
$60,000 to under $100,000	1,260 (24.2)	338 (31.3)	922 (68.7)	
$100,000 or more	1,365 (28.0)	416 (34.6)	949 (65.4)	
Missing	40 (0.9)	20 (55.3)	20 (44.7)	
Type of community				
Urban	2,260 (36.9)	537 (26.9)	1723 (73.1)	0.005
Suburban	2,311 (48.8)	666 (33.1)	1,645 (66.9)	
Rural	740 (14.3)	207 (30.7)	533 (69.3)	
High neighborhood disorder				
No	3,781 (73.0)	1,058 (32.5)	2,723 (67.5)	<0.001
Yes	1,530 (27.0)	352 (24.8)	1,178 (75.2)	
Poor home conditions				
No	4,107 (78.1)	1,223 (34.4)	2,884 (65.6)	<0.001
Yes	1,204 (21.9)	187 (16.3)	1,017 (83.7)	
Not having enough money to pay bills				
No	4,386 (82.5)	1,161 (30.1)	3,225 (69.9)	0.36
Yes	925 (17.5)	249 (32.3)	676 (67.7)	
Skip meals or eat less because did not have enough money for food				
No	4,163 (79.3)	1,140 (31.6)	3,023 (68.4)	0.01
Yes	1,148 (20.7)	270 (26.1)	878 (73.9)	
Last saw dentist more than 2 years ago (or never)				
No	4,209 (79.3)	1,208 (33.3)	3,001 (66.7)	<0.001
Yes	1,102 (20.7)	202 (19.3)	900 (80.7)	
Non-victimization adversity: M (SE)	2.12 (0.04)	1.02 (0.05)	2.61 (0.05)	<0.001

Weighted percentages, unweighted N. Row percentages.

a Also significantly different with missing values dropped.

### 2.2 Procedures

Randomly selected AmeriSpeak panelists were sent a description of the study by email, and an invitation to complete the survey. Incentives of $20 were provided for those participants who completed the survey. With over 55,000 U.S. residents, the AmeriSpeak panel is designed to be representative of the U.S. household population using probability-based sampling. Details of recruitment into the AmeriSpeak panel are published elsewhere (29). The survey completion rate among those sampled for this study was 33.0%.

NORC at the University of Chicago’s Institutional Review Board approved the project for data collection. The research team obtained voluntary and informed consent from all participants either by the participant consenting verbally for those completing a survey by phone or by clicking a time-stamped box for those completing the online version of the survey. Participants under the age of 18 provided assent after caregiver consent was obtained. Cognitive testing of the survey instrument (*n* = 5 with some youth under 18 years old and some over age 18), including questions about firearm exposures, helped ensure wording was appropriate for the age range of participants.

### 2.3 Measures

Exposure to firearm violence, threats, or risky firearm behavior. We developed a series of 10 questions for the current study that queried exposures to someone else’s firearm violence, threats and risky behaviors. Items were drawn from prior work about youth gun violence exposure which included focus groups and cognitive interviews with youth as young as age 10 (18). These were designed to expand upon previous measures that tend to focus mainly on seeing or being present when someone was shot (30) or census tract numbers of firearm homicides (16). Before these questions, participants were told that we were only asking about things they may have seen or heard about in real life—not things they may have seen on TV, in a movie, on the news or in a video game. We grouped these questions together based on the type of situation into the following five categories. The questions asked, “Have you ever…” (yes/no):

Seen someone shooting a gun in a public place (like on the street, or a parking lot, school, or store)?
Heard (but not seen) a gun being shot in a public place (like the streets, parking lots or stores)?
Firearm threats

- Heard or seen anyone you know talking or posting about hurting someone else with a gun?
- Seen someone or heard anyone threaten to hurt someone else with a gun?
Risky firearm access/possession
- Heard someone of any age talk about getting a gun or having a gun when they aren’t supposed to?
- Known someone of any age who had a gun when or where they were not supposed to?
- Known about anyone bringing a gun to school or work? Do not include situations where someone carried a gun to work because that was part of their job, like police or security officers.
- Been surprised or worried because someone you knew was carrying a gun (not for their job)?
Someone else’s self-directed firearm violence
- Heard or seen anyone you know talking or posting about hurting themselves with a gun?
- Known anyone who has killed or tried to kill themselves with a gun?

Variables were created to reflect any exposure within each of these five aggregate categories as well as exposure to any of the 10 items.

Demographic characteristics. Demographic characteristics measured include age, birth sex, gender identity, sexual identity, race, and ethnicity. Details of categories within each are reported in Table 1.

Social and structural determinants of health measures are usually drawn from secondary data sources (31) or involve screening tools designed for in-person assessments in clinical settings (32). The current measures were designed to build on these screening tools using self-report to document deficits social and structural determinants of health across four domains:

*Economic instability* includes 2 items, adapted from the Youth Risk Behavior Survey (32), for example: not having enough money to pay bills in the past 12 months? Response options ranged from 1 (never) to 5 (always).

*Social context* includes lifetime non-victimization adversity due to non-violent traumatic events and chronic stressors and was measured using 10 items (33) (for example, serious illnesses, accidents, family homelessness) which were combined into a count variable for current analyses (M = 2.12, SD = 2.15, *α* = 0.75).

*Health care.* Participants were asked, “When was the last time you saw a dentist for a check-up, exam, teeth cleaning, or other dental work?” (34). Response options were: in the past 12 months, between 1 and 2 years ago, more than 2 years ago, and I have never been to the dentist. This was coded into a new variable reflecting more than 2 years ago (or never) versus more recently.

*Neighborhood and built environment.*

Physical home environmental conditions included eight items adapted from the American Academy of Family Physicians Social Needs Screening Tool (35) covering problems where you currently live. Participants are told to think about their permanent place of residence, not a dorm room or other temporary housing and answer the following questions: bugs everywhere, mold, lead paint or lead pipes, not enough heat, the oven or stove does not work, there are no smoke detectors or they do not work, water leaks, frequent loss or no electricity. Response options for each were yes/no and summed to create a total count and then dichotomized at 1 standard deviation above the mean or higher to reflect poor home conditions.

Physical neighborhood environmental conditions included 12 items adapted from Perkins and colleagues (36) to be more meaningful to youth. Items measure residents’ perceptions of the severity of different neighborhood conditions. Participants were asked to rate each one as to whether it was: (0) no problem, (1) a minor problem, or (2) a serious problem in their neighborhood (“by neighborhood we mean the street you live on and a few streets around it”): for example, gangs, graffiti, drugs, homelessness. Items were summed to create a total neighborhood disorder score and then dichotomized at one standard deviation above the mean or higher to reflect high neighborhood disorder.

### 2.4 Data analysis

We applied statistical weighting to adjust the data to US census benchmarks to account for selection probabilities (balanced by age, sex, race/ethnicity, education, and region) and participant level non-response to the survey (using a response propensity approach calculating the conditional probability that a particular respondent completed the survey given observed covariates) (37). We derived the sampling weights using the final panel weight used in all AmeriSpeak studies and the probability of selection for the sampled panel members in our specific study on firearm violence (non-response adjusted).

Missing data were minimal (3% or less) and conservatively coded as “no exposure” for the main firearm violence questions. This amounts to 51 cases with missing data on the main firearm exposure measures. All analyses were also conducted with these 51 cases dropped and the results were the same both with these cases included and coded as “0” as they were when dropped. Missing data on four demographic characteristics are noted in Table 1 and these participants were dropped in multivariate analyses: sex at birth (*n* = 104), gender identity (*n* = 101), household income (*n* = 40), and race (*n* = 140) for a total n of 271 dropped from multivariate analyses.

Demographic and SDOH characteristics were compared by any exposure to firearm violence using two-way tabulations with tests of independence for complex survey designs. Then, prevalence rates and 95% confidence intervals are reported for different types of firearm violence exposure—both overall and for three age categories: 10–17 years olds, 18–24 year olds, and 25–34 year olds. Next, using logistic regression analyses, we present adjusted odds ratios for different demographic and SDOH characteristics for exposure to each of the five aggregate types of firearm exposure: (1) saw a shooting in a public place, (2) interpersonal firearm violence threats, (3) heard (but did not see) firearm shots in a public place, (4) knowledge of inappropriate access/possession of firearms, and (5) knowledge of someone’s self-directed firearm violence.

## 3 Results

Seven in 10 participants (69.5% of the sample) reported any exposure to firearm violence, threats or risky behavior in their lifetime using the 10-item definition as fielded in this study (Table 1). Significant demographic differences for any firearm violence exposure were noted. Exposure was more common among older participants, females or those intersex at birth, participants identifying as gender minority, sexual minority, Black or African American, American Indian or Alaska Native, or as two or more races. Different structural and social factors were also significantly related to firearm violence exposure—namely, living in lower income households, in urban communities, in neighborhoods self-classified as having high disorder, in a home with poor living conditions; having to skip meals or eat less due to a lack of money for food, lack of dental care, and non-victimization adversity.

### 3.1 Prevalence and types of lifetime firearm violence exposures—overall and by age

**Figure 1:**
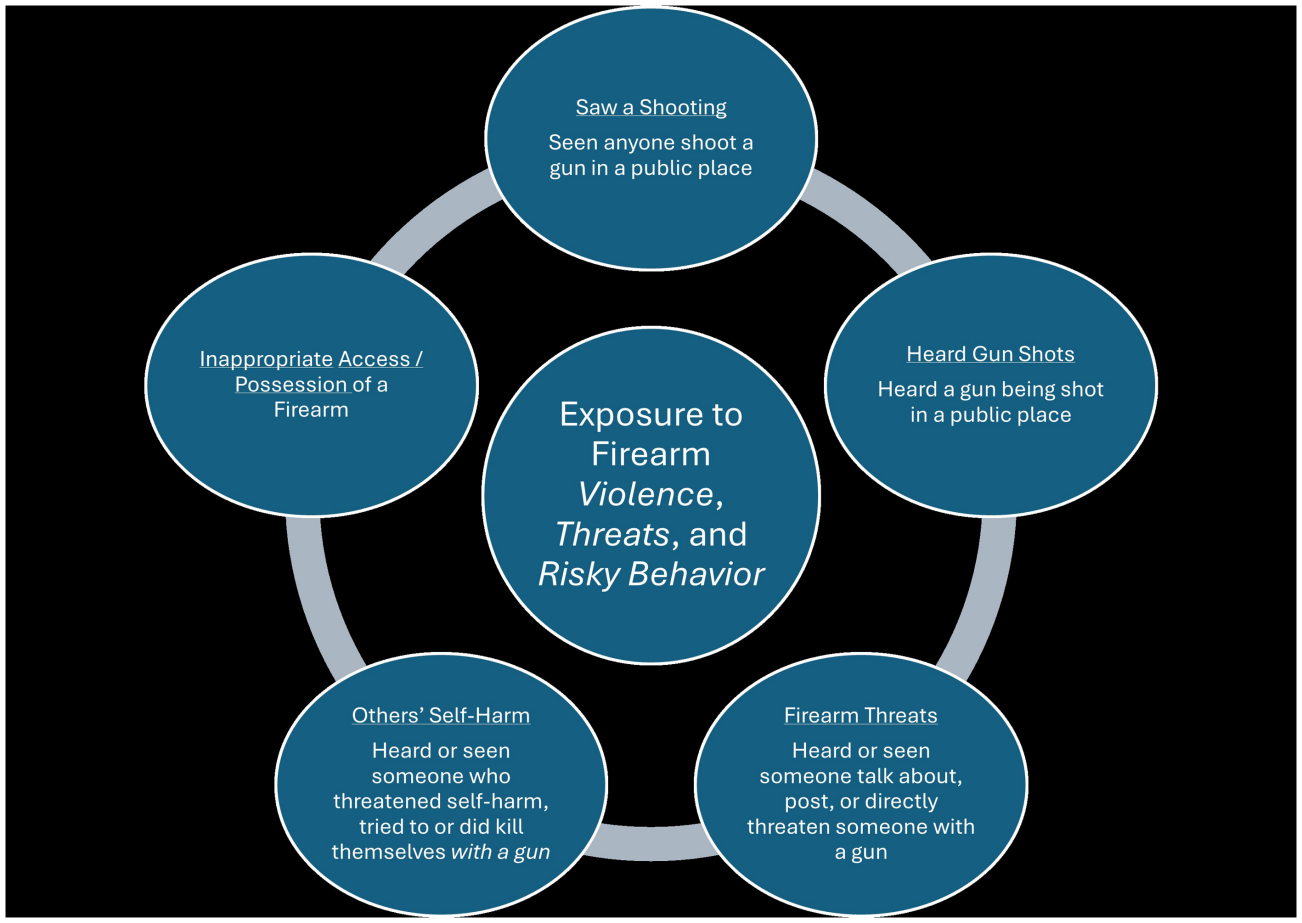
Ways Young People are Exposed to Firearm Violence, Threats, and Risky Behavior.

As depicted in Figure 1, lifetime exposure to firearm violence took various forms which were grouped into five main categories: (1) seeing a shooting in a public place (12.5%), (2) hearing (but did not seeing) firearm shots in a public place (45.4%), (3) exposure to interpersonal firearm violence threats (24.7%), (4) risky access/possession of firearms (46.7%), and (5) someone else’s self-directed firearm violence (27.0%). Rates for specific types of exposures within each of these categories are detailed in Table 2.

**Table 2:** Prevalence rates and 95% confidence intervals for different types of exposure to gun violence, threats, and risky behaviors.

Type of firearm violence exposure	All participants n (%)	SE	95% confidence interval
Saw someone shoot firearm in public place	771 (12.5)	0.6	11.3, 13.7
Heard (but did not see) firearm shots in public place	2,575 (45.4)	0.9	43.6, 47.2
Interpersonal firearm violence threats (any)	1,450 (24.7)	0.8	23.2, 26.3
Seen or heard someone threaten to hurt someone else with firearm	1,181 (19.7)	0.7	18.4, 21.2
Heard or seen anyone you know talking/posting about hurting someone else with firearm	705 (12.4)	0.6	11.2, 13.6
Risky access/possession of firearms (any)	2,670 (46.7)	0.9	44.9, 48.6
Heard someone talk about getting or having a firearm when they were not supposed to	1,514 (25.7)	0.8	24.2, 27.3
Known someone who had firearm when or where were not supposed to	1,545 (25.9)	0.8	24.4, 27.5
Surprised or worried because someone you know was carrying a firearm (not part of job)	1,027 (17.1)	0.7	15.8, 18.5
Known someone who brought firearm to school or work (not part of job)	1,212 (22.4)	0.8	20.9, 24.0
Someone else’s self-directed firearm violence (any)	1,540 (27.0)	0.8	25.5, 28.7
Heard or seen anyone you know talking/posting about hurting themselves with firearm	548 (9.9)	0.6	8.9, 11.1
Known someone who killed or tried to kill themselves with firearm	1,298 (21.9)	0.7	20.5, 23.4
Any of the above	3,901 (69.5)	0.9	67.8, 71.3

Lifetime exposure to each type of firearm violence significantly differed by age with the youngest participants (ages 10–17) being significantly less likely to report each experience than older participants. Prevalence rates and 95% confidence intervals by age are detailed in Table 3. The two older age groups (ages 18–24 and 25–34) were statistically similar to each other across all types of firearm violence exposure except for hearing but not seeing firearm shots, knowing someone who brought a firearm to school or work, and hearing or seeing someone they knew talking or posting about hurting themselves with a firearm which were more common among the 18–24 age group compared to the 25–34 group. The oldest age group was significantly more likely than the 18–24 participants to know someone who killed or tried to kill themselves with a firearm.

**Table 3:** Prevalence rates and 95% confidence intervals for different types of exposure to gun violence, threats, and risky behaviors by age category.

Type of gun violence exposure	10–17 year olds (*n* = 1,189)			18–24 year olds (*n* = 853)			25–34 year olds (*n* = 3,269)		
	n (%)	SE	95% CI	n (%)	SE	95% CI	n (%)	SE	95% CI
Saw someone shoot a gun in a public place	106 (7.1)	0.9	5.5, 9.1	143 (15.4)	1.5	12.6, 18.6	522 (14.4)	0.7	13.0, 16.0
Heard (but did not see) gun shots in a public place	383 (27.1)	1.6	24.1, 30.3	476 (56.3)	2.1	52.1, 60.4	1716 (51.3)	1.1	49.1, 53.4
Interpersonal gun threats (any)	205 (14.1)	1.2	11.8, 16.6	270 (29.3)	1.9	25.7, 33.2	975 (29.3)	1.0	27.3, 31.3
Saw or heard someone threaten to hurt someone else with a gun	147 (9.7)	1.0	7.9, 11.9	213 (23.1)	1.8	19.8, 26.7	821 (24.7)	0.9	22.9, 26.6
Heard or saw anyone you know talking/posting about hurting someone else with a gun	121 (8.7)	1.0	6.9, 10.9	141 (15.2)	1.5	12.5, 18.4	443 (13.1)	0.7	11.7, 14.6
Risky access/possession of guns (any)	396 (28.9)	1.6	25.8, 32.2	488 (55.5)	2.1	51.3, 59.7	1786 (53.7)	1.1	51.6, 55.8
Heard someone talk about getting or having a gun when they were not supposed to	228 (15.3)	1.2	13.1, 17.9	275 (30.3)	1.9	26.7, 34.2	1,011 (30.2)	1.0	28.3, 32.2
Knew someone who had a gun a when or where were not supposed to	180 (12.7)	1.2	10.5, 15.2	278 (30.4)	1.9	26.7, 34.3	1,087 (32.4)	1.0	30.4, 34.4
Knew someone who brought a gun to school or work (not part of job)	174 (12.9)	1.2	10.6, 15.5	260 (29.9)	1.9	26.3, 33.8	778 (24.3)	0.9	22.5, 26.1
Surprised or worried because someone you knew was carrying a gun (not part of job)	106 (8.5)	1.0	6.7, 10.8	187 (20.6)	1.7	17.5, 24.1	734 (21.0)	0.9	19.3, 22.7
Someone else’s self-directed gun violence (any)	164 (12.7)	1.2	10.5, 15.4	280 (32.8)	2.0	29.0, 36.9	1,096 (33.5)	1.0	31.5, 35.5
Heard or saw anyone you knew talking/posting about hurting themselves with a gun	71 (5.3)	0.8	3.9, 7.2	122 (14.0)	1.4	11.4, 17.1	355 (10.5)	0.7	9.2, 11.9
Known someone who killed or tried to kill themselves with a gun	120 (8.9)	1.0	7.1, 11.1	216 (24.9)	1.8	21.5, 28.7	962 (29.3)	1.0	27.4, 31.2
Any of the above	638 (48.0)	1.8	44.4, 51.6	685 (80.3)	1.7	76.7, 83.4	2,578 (77.9)	0.9	76.1, 79.7

### 3.2 Adjusted odds of five different types of firearm violence exposures by demographic characteristics and SDOH deficits

The odds of having ever *seen someone shoot a firearm in a public place* were significantly higher for older participants compared to participants aged 10–17 years (see Table 4). Black or African American participants and American Indian or Alaska Native had higher odds in comparison to White participants of seeing a gun being shot in a public place. Participants living in poor home conditions, in neighborhoods with high disorder, and having to skip meals or eat less do a lack of money were more likely to have seen a shooting compared with those who did not live under such conditions. For each additional non-victimization adversity indicated, there was a 1.23 increase in odds of having seen a shooting. Lifetime *exposure to interpersonal firearm violence threats* was higher for sexual minority, Black or African American, American Indian or Alaska Native, and those who identified with two or more races compared to participants without that racial identity, respectively. Living in an area with high neighborhood structural disorder and experiencing non-victimization adversity were associated with elevated odds of lifetime exposure to firearm violence threats. *Having heard (but not seen) firearm shots in a public place* was higher for Black or African American participants, those living in poor home or neighborhood conditions, and those who indicated experiences of non-victimization adversity.

**Table 4:** Adjusted odds of lifetime exposure to interpersonal firearm threats and shots/shootings by participant demographic and structural and social characteristics.

	Saw someone shoot a firearm in public place		Exposure to interpersonal firearm threats		Heard (but did not see) firearm shots in public place	
	Odds ratio (95% CI)	*p* value	Odds ratio (95% CI)	*p* value	Odds ratio (95% CI)	*p* value
Demographic						
Gender and sexual identity						
Female (ref)	1.0		1.0		1.0	
Male	1.13 (0.89, 1.43)	0.32	1.11 (0.92, 1.35)	0.28	1.05 (0.89, 1.24)	0.54
Gender minority	1.63 (0.86, 3.10)	0.13	1.54 (1.00, 2.39)	0.05	1.72 (1.09, 2.74)	0.02
Sexual minority	0.96 (0.73, 1.27)	0.77	1.29 (1.02, 1.61)	0.03	1.14 (0.91, 1.41)	0.25
Age						
10–17 (ref)	1.0		1.0		1.0	
18–24	1.79 (1.21, 2.64)	0.004	1.80 (1.34, 2.43)	<0.001	2.84 (2.22, 3.65)	<0.001
25–34	1.44 (1.04, 2.00)	0.03	1.66 (1.30, 2.12)	<0.001	2.03 (1.67, 2.48)	<0.001
Race/Ethnicity						
White (ref)	1.0		1.0		1.0	
Black or African American	2.75 (2.04, 3.70)	<0.001	2.48 (1.94, 3.17)	<0.001	1.91 (1.53, 2.40)	<0.001
Asian	1.27 (0.77, 2.09)	0.35	1.15 (0.83, 1.58)	0.39	0.81 (0.61, 1.05)	0.12
American Indian or Alaska Native	2.88 (1.13, 7.32)	0.03	2.60 (1.10, 6.13)	0.03	1.83 (0.85, 3.94)	0.12
Native Hawaiian	0.23 (0.03, 1.84)	0.17	0.74 (0.16, 3.34)	0.69	1.26	0.69
Other race	1.00 (0.58, 1.70)	0.99	1.17 (0.74, 1.85)	0.51	1.25 (0.84, 1.85)	0.27
Two or more races	1.30 (0.86, 1.96)	0.21	1.78 (1.30, 2.44)	<0.001	1.26 (0.93, 1.71)	0.13
Hispanic ethnicity	1.33 (0.97, 1.81)	0.07	0.87 (0.67, 1.14)	0.33	0.99 (0.79, 1.24)	0.90
Structural and Social						
Type of community						
Urban (ref)	1.0		1.0		1.0	
Suburban	0.70 (0.54, 0.91)	0.006	0.89 (0.73, 1.09)	0.26	0.77 (0.65, 0.91)	0.003
Rural	0.85 (0.59, 1.23)	0.39	0.92 (0.68, 1.23)	0.57	0.63 (0.48, 0.83)	0.001
Household Income						
Less than $30,000 (ref)	1.0		1.0		1.0	
$30,000 to <$60,000	1.35 (1.00, 1.81)	0.05	1.37 (1.06, 1.76)	0.01	1.37 (1.09, 1.73)	0.008
$60,000 to under $100,000	1.04 (0.74, 1.45)	0.83	1.27 (0.96, 1.68)	0.09	1.07 (0.83, 1.38)	0.59
$100,000 or more	1.15 (0.79, 1.67)	0.46	1.27 (0.95, 1.71)	0.11	1.12 (0.87, 1.44)	0.37
High neighborhood disorder	1.73 (1.32, 2.27)	<0.001	1.49 (1.20, 1.85)	<0.001	1.48 (1.21, 1.81)	<0.001
Poor home conditions	1.34 (1.01, 1.78)	0.04	1.16 (0.93, 1.45)	0.18	1.24 (1.01, 1.53)	0.04
Not having enough money to pay bills	0.87 (0.64, 1.18)	0.36	1.02 (0.79, 1.31)	0.86	0.87 (0.69, 1.11)	0.25
Skip meals or eat less because did not have enough money for food	1.40 (1.03, 1.91)	0.03	1.21 (0.94, 1.55)	0.13	0.82 (0.64, 1.04)	0.10
Last saw dentist more than 2 years ago (or never)	1.21 (0.93, 1.57)	0.16	1.07 (0.85, 1.34)	0.57	1.20 (0.96, 1.50)	0.11
Non-victimization adversity	1.23 (1.17, 1.29)	<0.001	1.33 (1.27, 1.38)	<0.001	1.30 (1.25, 1.35)	<0.001

*N* = 271 participants dropped from the multivariate analysis due to missing data on sex at birth, gender, income and/or race. ***p ≤ 0.001, **p ≤ 0.01, *p ≤ 0.05. Ref, reference group.

Similar patterns were noted for elevated odds of lifetime *exposure to risky firearm carrying or possession,* with the additional elevated odds for respondents living in rural communities and living in higher income households (Table 5). *Knowledge of someone else’s self-directed violence with a firearm* did not show the same racial identity differences as the other types of exposure, i.e., this form of exposure over respondents’ lifetimes was distributed similarly across individuals of different racial identity. However, elevated odds of knowing someone at risk of self-directed firearm violence were identified for sexual minority participants and those living in rural communities. Odds of lifetime exposure to someone else’s self-directed firearm violence were also higher for those living in higher income households. Hispanic participants were significantly less likely than non-Hispanic participants to have known someone who had or was thinking about using a firearm for self-harm. Living in poor neighborhood conditions, having to skip meals due to a lack of money, and non-victimization adversity were also associated with increased lifetime odds of this type of exposure.

**Table 5:** Adjusted odds of lifetime knowledge of inappropriate firearm carrying / possession and someone else’s self-directed violence by participant demographic and structural and social characteristics.

	Knowledge of inappropriate firearm carrying / possession		Knowledge of self-directed firearm violence	
	Odds ratio (95% CI)	*p* value	Odds ratio (95% CI)	*p* value
Demographic				
Gender and sexual identity				
Female (ref)	1.0		1.0	
Male	1.03 (0.87, 1.21)	0.75	0.95 (0.79, 1.15)	0.59
Gender minority	1.33 (0.79, 2.23)	0.28	0.77 (0.49, 1.21)	0.26
Sexual minority	1.24 (0.99, 1.55)	0.07	1.27 (1.02, 1.58)	0.03
Age				
10–17 (ref)	1.0		1.0	
18–24	2.44 (1.90, 3.14)	<0.001	2.91 (2.15, 3.94)	<0.001
25–34	2.07 (1.70, 2.53)	<0.001	2.79 (2.15, 3.62)	<0.001
Race/Ethnicity				
White (ref)	1.0		1.0	
Black or African American	1.73 (1.38, 2.18)	<0.001	1.01 (0.78, 1.30)	0.95
Asian	0.73 (0.56, 0.96)	0.03	0.52 (0.36, 0.75)	<0.001
American Indian or Alaska Native	2.04 (0.95, 4.37)	0.07	0.84 (0.29, 2.43)	0.75
Native Hawaiian	0.28 (0.06, 1.20)	0.09	0.32 (0.06, 1.56)	0.16
Other race	1.24 (0.83, 1.85)	0.29	0.84 (0.50, 1.41)	0.51
Two or more races	1.50 (1.12, 2.00)	0.006	1.04 (0.76, 1.43)	0.79
Hispanic ethnicity	0.96 (0.76, 1.21)	0.73	0.67 (0.51, 0.89)	0.005
Structural and Social				
Type of community				
Urban (ref)	1.0		1.0	
Suburban	0.90 (0.76, 1.08)	0.26	0.92 (0.76, 1.12)	0.43
Rural	1.34 (1.03, 1.74)	0.03	1.86 (1.41, 2.44)	<0.001
Household Income				
Less than $30,000 (ref)	1.0		1.0	
$30,000 to <$60,000	1.31 (1.03, 1.66)	0.03	1.29 (1.01, 1.66)	0.04
$60,000 to under $100,000	1.32 (1.03, 1.70)	0.03	1.63 (1.23, 2.15)	0.001
$100,000 or more	1.47 (1.13, 1.90)	0.004	1.73 (1.30, 2.29)	<0.001
High neighborhood disorder	1.21 (0.99, 1.49)	0.07	1.49 (1.20, 1.85)	<0.001
Poor home conditions	1.53 (1.24, 1.89)	<0.001	1.04 (0.83, 1.30)	0.72
Not having enough money to pay bills	1.04 (0.82, 1.32)	0.77	0.88 (0.67, 1.15)	0.36
Skip meals or eat less because did not have enough money for food	1.11 (0.87, 1.41)	0.39	1.29 (1.00, 1.66)	0.05
Last saw dentist more than 2 years ago (or never)	1.04 (0.84, 1.29)	0.73	0.98 (0.78, 1.23)	0.87
Non-victimization adversity: M (SE)	1.33 (1.27, 1.39)	<0.001	1.28 (1.22, 1.33)	<0.001

*N = 271 participants dropped from the multivariate analysis due to missing data on sex at birth, gender, income and/or race. Ref, reference group.*

## 4 Discussion

Findings from the *Growing up With Guns Study* offers one of the first assessments of the prevalence of lifetime exposure to a wide range of different situations involving firearm violence, threats, and risky behavior in a general population sample from the United States. Rates of exposure were high across age categories, ranging from 48% of 10–17 year olds to 80.3% of 25–34 year olds. Importantly, the measures of exposure were broad, allowing for the study of an array of experiences including bystander, vicarious, and community roles, as described by Bancalari and colleagues (15), and also including exposure to someone else’s threat or use of guns for self-directed violence. This is a significant measurement contribution of the current study as it builds on previous critiques and recommendations. Findings from this population-based, nationally representative data provide insights into future opportunities for the development of primary and secondary prevention and intervention efforts that may be applicable to a broad spectrum of youth and young adults who represent high-risk for firearm violence exposures across different developmental age groups.

The current study revealed high rates (more than 4 in 10 participants) of having seen shooting in a public place, the closest proximity to firearm violence exposure measured in the current study. This is a scenario which could place people who are bystanders in physical danger. Data also revealed high rates of hearing firearm shots in public places, including almost three in 10 youth; a situation found to have negative consequences especially for young children (38). These are the more well-researched forms of firearm violence exposure. The current study, however, also found high rates of exposure (one in four participants) to visual or written interpersonal firearm violence threats and almost one in two participants had knowledge of risky access to or possession of a firearm. These situations present potential opportunities to prevent shootings as bystanders can call for help and potentially keep firearms from being used. This is consistent with recent studies showing a difference between completed school shootings and those that were averted due to reports by friends and acquaintances (39).

The current study also highlighted both high rates of exposure to someone else’s firearm use in suicidal behaviors. One in five participants knew someone who had killed or tried to kill themselves with a firearm and 9.9% had heard or seen someone they know talking or posting on social media about hurting themselves with a firearm. This is critical from a public health perspective as the likelihood of death by suicide is increased when someone has access to a firearm (40, 41). This data suggests another potential point of bystander intervention when friends work to connect at-risk peers to crisis hotlines and services. Understanding and building strengths among different groups of young people to educate them on alternatives to firearm use is an important investment for prevention.

Exposure to firearm violence is not evenly distributed across social identity groups. Previous research has found differential risk for firearm violence by demographic variables including age, race, and gender (42–44). Research also indicates place-based risk factors including urban and rural locations, and community variables such as poverty and crime rates (42, 45, 46). The current study also documented demographic group differences. Specifically, sexual minority participants had greater odds of firearm violence exposure overall. This was not only for use of firearms in incidents of self-directed violence, which is consistent with the higher risk of self-directed violence among sexual minority communities (47), but also elevated exposure to interpersonal firearm violence threats. Similarly, Black and African American and American Indian or Alaska Native had elevated odds of exposure to interpersonal firearm violence, threats or risky behavior. African American children have the highest rates of firearm mortality overall (5.7 per 100,000) (48) and the current study suggests this is true of exposure to firearm violence as well. Findings are in line with national data on firearm violence exposure among Black, as well as American Indian or Alaska Native adults (49). Firearm violence is yet another stressor that contributes to the burden of health disparities faced by these communities (50, 51), communities which often already have higher rates of depression and anxiety (52, 53). Healthcare systems, including emergency rooms, are increasingly playing a role in identifying firearm violence risk and may be an important point of intervention for these groups who have also faced historic and systematic oppression and discrimination.

Findings also highlight the importance of taking a life course perspective to better prevent and intervene in different types of firearms exposures. A developmental or life course perspective highlights the importance of considering how age-related life stages—and characteristics of social interaction, situational contexts, and choice-making over time (54)–may shape risk and protective factors for firearm violence. A life course perspective focuses on connections between adolescence and two critical developmental time periods surrounding it–both childhood and young adulthood (55). Underlying this perspective is the idea that no developmental stage can be understood in isolation from others. Indeed, social factors in childhood influence the processes of development and are the beginning of socially determined pathways to health and behavior in later life (56). Such a perspective can also serve as a tool for understanding health disparities across different vulnerable populations of youth (57). Not surprisingly, the adolescents in the current study were less likely to report firearm violence exposures compared with the young adults, given this was a lifetime rate and thus they had less opportunity for exposure. At the same time, 48% of these adolescents reported at least one type of exposure to firearm violence, threats, or risky behaviors.

A key finding of prior research is how cumulative exposures to victimization and other adversities, like firearm exposures, lead to problematic developmental outcomes. Key concepts that have emerged is that of the “poly-victim,” youth who suffer a growing disproportionate quantity of serious victimization and a much greater array and intensity of negative effects (58–61) and adverse childhood experiences (62, 63) with a linear relationship between the accumulation of adversity types and the level of adverse outcomes (64). Firearm factors may play into the adversity accumulation cycle in various ways. Negative firearm exposures, for example, may be particularly salient or traumatizing contributions to the cycle. Firearm fascination, acquisition and carrying may be a response among highly exposed children and youth, which may in turn aggravate the cycle. It is critical to continue to build our knowledge of the range of firearm exposures for youth and to collect data on the contexts and subgroups of youth for whom these exposures are related to greater risk of harm over time.

Social and structural determinants of health are conditions in the places where people live, learn, work, and play that affect a wide range of health and health indicators (21). There is growing awareness of the importance of including assessments of SDOH as they help overcome the limitations of assessing only demographic markers (22). The current study found elevated odds of firearm violence exposure for those living in poor home conditions, in neighborhoods with high disorder, and experiencing economic instability, dental care constraints, and non-victimization adversity. This is consistent with community-level and community-focused studies of firearm violence that seek to move beyond individual risk and protective factors (13). Awareness of firearms and related violence, threats and risky behavior may be a response to safety concerns in neighborhoods that are systematically under-resourced by policies and systemic practices that sustain chronic lack of investment in some communities. These findings remind us that interventions need to be inclusive of settings, not just people.

### 4.1 Limitations

Findings should be considered in the context of some limitations. Our data are cross-sectional and we are thus unable to make causal statements about our findings. The lifetime measures of firearm violence exposure across age groups are differentiated by opportunities of exposure based on time; we cannot discern if there are actual cohort effects. Data are self-reported and thus susceptible to under- or over-reporting of responses. The measures for structural conditions in this study were based on current experiences and may not reflect the community in which the participant became aware of the firearm violence situation.

## 5 Conclusion

The current findings suggest some promise for innovative prevention and intervention strategies that move beyond victims and perpetrators of firearm violence to engage bystanders. The study underscores ways that exposures to someone else’s firearm violence, threats, and risky behaviors are associated with low resources associated with SDOH deficits. Prevention and intervention strategies need to change communities, and especially access to resources, not just individual attitudes and behaviors. A more thorough contextual understanding of firearm violence exposures, including relationships among the people involved, different ways people intervene, and barriers to intervention, are critical next steps that can help guide the development of new bystander interventions targeting firearm violence.

## Data Availability

The raw data supporting the conclusions of this article will be made available by the authors, without undue reservation.
